# Deletion of the EP296R Gene from the Genome of Highly Virulent African Swine Fever Virus Georgia 2010 Does Not Affect Virus Replication or Virulence in Domestic Pigs

**DOI:** 10.3390/v14081682

**Published:** 2022-07-30

**Authors:** Elizabeth A. Vuono, Elizabeth Ramirez-Medina, Sarah Pruitt, Ayushi Rai, Nallely Espinoza, Edward Spinard, Alyssa Valladares, Ediane Silva, Lauro Velazquez-Salinas, Manuel V. Borca, Douglas P. Gladue

**Affiliations:** 1 Plum Island Animal Disease Center, ARS, USDA, Greenport, New York, NY 11944, USA; elizabeth.vuono@usda.gov (E.A.V.); elizabeth.ramirez@usda.gov (E.R.-M.); sarah.pruitt@usda.gov (S.P.); ayushi.rai@usda.gov (A.R.); nallely.espinoza@usda.gov (N.E.); edward.spinnard@usda.gov (E.S.); alyssa.valadares@usda.gov (A.V.); ediane.silva@usda.gov (E.S.); lauro.valazquez@usda.gov (L.V.-S.); 2 Department of Pathobiology and Population Medicine, Mississippi State University, P.O. Box 6100, Oxford, MS 39762, USA; 3 Oak Ridge Institute for Science and Education (ORISE), Oak Ridge, TN 37830, USA

**Keywords:** ASFV, ASF, African swine fever, African swine fever virus, EP296R, virus virulence

## Abstract

African swine fever virus (ASFV) causes a lethal disease (ASF) in domestic pigs, African swine fever (ASF). ASF is currently producing a pandemic affecting pig production across Eurasia, leading to a shortage of food accessibility. ASFV is structurally complex, harboring a large genome encoding over 150 genes. One of them, EP296R, has been shown to encode for an endonuclease that is necessary for the efficient replication of the virus in swine macrophages, the natural ASFV target cell. Here, we report the development of a recombinant virus, ASFV-G-∆EP296R, harboring the deletion of the EP296R gene from the genome of the highly virulent field isolate ASFV Georgia 2010 (ASFV-G). The recombinant ASFV-G-∆EP296R replicates in primary swine macrophages with similar kinetics as the parental virus ASFV-G. Pigs experimentally infected by the intramuscular route with 10^2^ HAD_50_ show a slightly protracted, although lethal, presentation of the disease when compared to that of animals inoculated with parental ASFV-G. Viremia titers in the ASFV-G-∆EP296R-infected animals closely followed the kinetics of presentation of clinical disease. Results presented here demonstrate that ASFV-G-∆EP296R is not essential for the processes of ASFV replication in swine macrophages, nor is it radically involved in the process of virus replication or disease production in domestic pigs.

## 1. Introduction

African swine fever is a lethal disease of domestic pigs geographically expanding as a pandemic affecting countries across Eurasia, severely damaging their swine production industry. The disease reappeared in the Dominican Republic and Haiti after more than 40 years of being absent in the Western hemisphere [1]. The first commercial vaccine was recently approved for use in Vietnam, containing a single deletion in the I177L gene [2]. However, in other countries where there is no commercial vaccine available to prevent the disease, control is limited to culling all infected animals and trying to limit animal mobilization in the affected geographical area to try and contain the disease [3].

The etiological agent of ASF, ASF virus (ASFV), is a highly structurally complex virus harboring a large double-stranded DNA genome (180–190 kb) which encodes more than 150 genes [4]. The functions of many of these genes are still unidentified or only have a predicted function. Discovering the function of several genes was useful in the discovery of live-attenuated vaccine candidates [2,5,6,7,8,9,10,11]. Deletion of virus genes involved in disease production allowed the rational development of recombinant attenuated virus strains that efficaciously protect against the lethal infection with the parental virulent virus [3]. In the same way, the discovery of virus genes implicated in critical steps in the process of virus replication might provide crucial evidence for the potential development of therapeutic tools to inhibit or restrict virus infection. One of those genes, EP296R, has been experimentally shown to have both endonuclease activity and proof-reading exonuclease activity [12]. In addition, EP296R gene function appears to be critical in the process of virus replication. A recombinant virus harboring the deletion of the gene from the genome of the ASFV BA71V strain, a virus stain adapted to grow in the Vero cell line, showed similar results in Vero cells [12].

Here, we report that a recombinant virus, ASFV-G-∆EP296R, a derivative of the highly virulent Georgia 2010 isolate lacking the EP296R gene, replicates in primary swine macrophage cultures as efficiently as the parental virus ASFV-G does. Pigs experimentally infected with ASFV-G-∆EP296R showed a fatal but slightly protracted form of the disease when compared to that of animals inoculated with parental ASFV-G. Viremia titers in the ASFV-G-∆EP296R-infected animals were closely associated with the progression of the severity of the clinical disease. Results presented here demonstrate that ASFV-G-∆EP296R is not essential for the processes of ASFV replication in swine macrophages nor critically implicated in the process of virus virulence in domestic pigs.

## 2. Materials and Methods

### 2.1. Viruses and Cells

Primary swine macrophages cultures were produced as previously described [13], and cell culture plates were seeded at a density of 5 × 10^6^ cells per mL. ASFV Georgia (ASFV-G) was a field isolate kindly provided by Dr. Nino Vepkhvadze from the Laboratory of the Ministry of Agriculture (LMA) in Tbilisi, Republic of Georgia [14]. Comparative growth curves between ASFV-G-∆EP296R and ASFV-G were performed on primary swine macrophages in triplicate, using six-well plates with an MOI of 0.01 HAD_50_ (hemadsorbing doses) as previously described [15]. Sample points were taken at 2, 24, 48, 72, and 96 h, cells were frozen at ≤−70 °C and thawed, and the lysates were titrated by HAD_50_/_mL_ in primary swine macrophage cell cultures in 96-well plates. The presence of the virus in infected cells was assessed by hemadsorption (HA), and virus titers were calculated as previously described [16].

### 2.2. Detection of EP296R Transcription

As previously described [17] we used a real-time PCR assay (qPCR) to evaluate the transcriptional profile of the EP296R gene during the infection of ASFV-G in cultures of porcine macrophages, using the early CP204L (p30) and late B646L (p72) expressed genes of ASFV as reference genes. Briefly, cell cultures of porcine macrophages were infected with a stock of ASFV-G using an MOI of 1. RNA extractions using an RNeasy Kit (QIAGEN, Hilden, Germany) were conducted at 0, 4, 6, 8, and 24 h post infection. All extractions were treated with two units of DNase I (BioLabs, San Diego, CA, USA), and then purified using the Monarch^®^ RNA Cleanup Kit (New England BioLabs, Inc., Ipswich, MA, USA). One microgram of RNA was used to produce cDNA using qScript cDNA SuperMix (Quanta bio, Beverly, MA, USA) that was used for the qPCR. Primers and probes for the detection of the EP296R gene were designed using the ASFV Georgia 2007/1 strain (GenBank Accession #NC_044959.2): forward, 5′–GAACCTACTTAGATGTGCCCTG–3′; reverse, 5′–ACCCTTTAATACCGACTTCCTTG–3′; probe, 50–FAM–TGTAACAAACGCACTCTTACGGGACC–MGBNFQ–3′. Primers and probes for the detection of p72 gene were as follows: forward, 5′–CTTCGGCGAGCGCTTTATCAC–3′; reverse, 5′–GGAAATTCATTCACCAAATCCTT–3′; probe, 5′–FAM–CGATGCAAGCTTTAT–MGB NFQ–3′. Primers and probes for the detection of CP204L (p30) gene were as follows: forward, 5′–GACGGAATCCTCAGCATCTTC–3′; reverse, 5′–CAGCTTGGAGTCTTTAGGTACC–3′; probe, 5′–FAM–TGTTTGAGCAAGAGCCCTCATCGG–MGBNFQ–3′. Primers and probes for the detection of the β-actin gene were as follows: forward, 5′–GACCTGACCGACTACCTCATG–3′; reverse, 5′–TCTCCTTGATGTCCCGCAC–3′; probe, 5′–FAM–CTACAGCTTCACCACCACGGC–MGBNFQ–3′. All qPCRs were conducted using the TaqMan Universal PCR Master Mix (Applied Biosystems, Waltham, MA, USA) using the following amplification conditions: one step at 55 °C for 2 min, followed by one denaturation step at 95 °C for 10 min, then 40 cycles of denaturation at 95 °C for 15 s, and annealing/extension at 65 °C for 1 min.

### 2.3. Construction of the ASFV EP296R Deletion Mutant

A recombinant mutant virus harboring a deletion of the EP296R gene (ASFV-G-∆EP296R) was developed after homologous recombination of the parental virus (ASFV-G) genome and the recombination transfer vector p72mCherryΔEP296R, as previously described [10]. p72mCherryΔEP296R contains the genomic regions adjoining the EP296R gene: the left arm located between genomic positions 168,173 and 169,173, and the right arm located between genomic positions 169,867 and 170,867. p72mCherryΔEP296R also contains a reporter gene cassette containing the mCherry fluorescent protein (mCherry) gene under the control of the ASFV p72 late gene promoter [18]. The p72mCherryΔEP296R vector was obtained by DNA synthesis (Epoch Life Sciences, Sugar Land, TX, USA). The designed construct produced a 693-nucleotide deletion between nucleotide positions 169,174 and 169,866, leaving only 198 nucleotides in the C-terminus of EP296R to not interfere with the promoter of E111R. This remaining portion of EP296R has no promoter; therefore, it is unlikely to be expressed. The recombinant ASFV-G-∆EP296R was purified by consecutive limiting dilution steps based on mCherry activity detection and full-length sequenced using next-generation sequencing (NGS).

### 2.4. Next-Generation Sequencing of ASFV

Virus DNA was extracted from infected macrophage cultures presenting higher than 90% CPE. The nucleus and cytoplasmic fractions were separated using the nuclear extraction kit with the viral DNA being isolated from the cytoplasmic fraction following the manufacturer’s protocol (Active Motif cat# 40010). ASFV-infected cells were treated with the hypotonic buffer on ice until the cell membrane was dissolved. The nucleus fraction was separated by centrifugation, the cytoplasmic fraction was collected, and DNA was extracted by adding 10% (*v*/*v*) of 3 M Na0Ac (Sigma-Aldrich, Rockville, MD, USA, 71,196) and an equal volume of phenol, chloroform, and isoamyl alcohol (25:24:1) with a pH of 6.5–6.9 (Sigma-Aldrich P3803–100ML). The preparation was centrifuged for max speed in a tabletop centrifuge; the aqueous layer was then ethanol-precipitated using two volumes of 100% ethanol, washed with the same volume of 70% ethanol, and dried. The resulting DNA pellet was then reconstituted in sterile water. We then used this DNA library for NGS using a Nextera XT kit in the NextSeq (Illumnia, San Diego, CA, USA) following the manufacturer’s protocol. Sequence analysis was performed using CLC Genomics Workbench software (CLCBio, Waltham, MA, USA).2.3.

### 2.5. Evaluation of ASFV-G-ΔEP296R Virulence in Domestic Pigs

The virulence of ASFV-G-∆EP296R was assessed in 35–40 kg commercial breed swine. Five pigs were intramuscularly (IM) inoculated with 10^2^ HAD_50_ of ASFV-G-∆EP296R. A similar group of animals were inoculated with 10^2^ HAD_50_ of ASFV-G. The appearance of clinical signs (anorexia, depression, fever, purple skin discoloration, staggering gait, diarrhea, and cough) and body temperature were monitored daily during the experiment. Blood samples were scheduled to be obtained at 0, 4, 7, 11, 14, 21, and 28 days post inoculation (pi) using tubes containing EDTA. Animal experiments were performed under biosafety level 3 conditions in the animal facilities at Plum Island Animal Disease Center, following a strict protocol approved by the Institutional Animal Care and Use Committee (number 225.06-19-R, approved 09-10-19).

## 3. Results

### 3.1. Genetic Diversity and Evolutionar Signatures of the EP296R Gene

**Figure 1:**
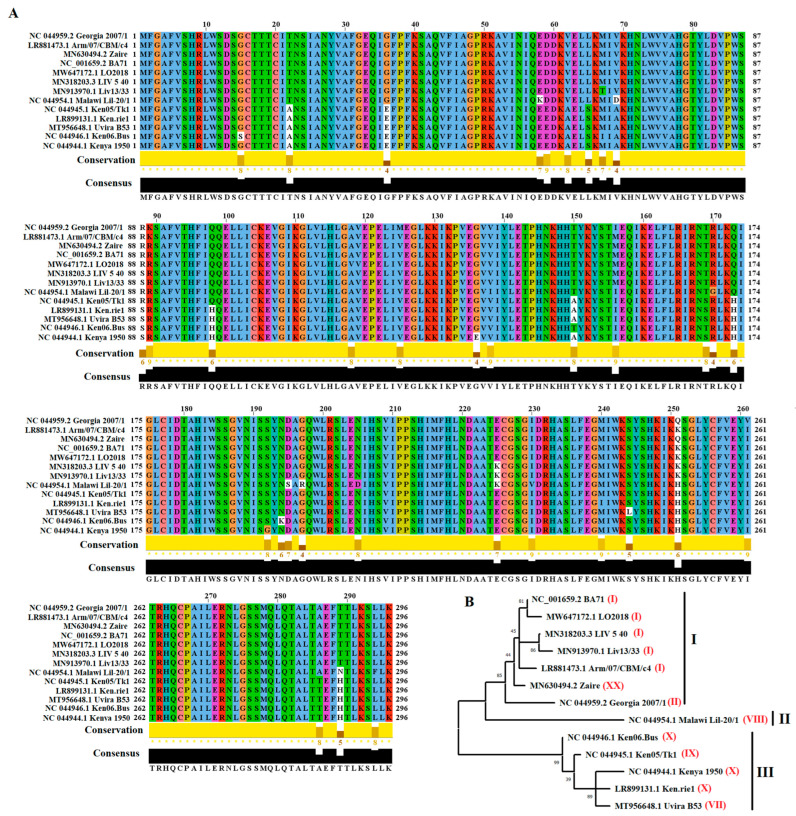
Evolution of EP296R protein among ASFV isolates. (**A**) Amino-acid alignment produced by the software Jalview version 2.11.1.7 showing the diversity of EP296R protein among a group of 13 representative ASFV isolates. Conservation plot scores reflected the nature of the change in specific sites, as high scores were associated with changes with similar biological properties. (**B**) Phylogenetic analysis conducted by the neighbor-joining method and the *p*-distance model, showing the diversity of EP296R protein of ASFV in the field. On the basis of the cluster distribution, isolates were classified in three main groups (I, II, and III). Numbers in the parenthesis (red), show the genetic lineage classification of different ASFV isolates based on p72 classification. Numbers above internal branches represent bootstrap values (1000 repetitions). Phylogenetic analysis was conducted using the software MEGA 10.2.5.

To evaluate the genetic diversity of EP296R gene of ASFV, a total of 116 nucleotide sequences were retrieved form GenBank database. After an initial screening using the software Jalview version 2.11.1.7, all redundant sequences were removed to obtain a final set of 13 viral sequences. This representative set of sequences includes viral isolates from genotypes I, II, VII, VIII, IX, X, and XX (Figure 1). To assess the levels of identity among isolates, we conducted a pairwise analysis using the *p*-distance model and the bootstrap method to give statistical confidence (*p* < 0.05). The results of the analysis showed an overall identity between 92.34% and 99.88% (−96.19%) and 91.89% and 99.66% (−91.89%) at nucleotide and amino-acid levels respectively, indicating the high conservation of EP296R gene among isolates of ASFV. In this context, we observed a high conservation of the EP296R gene among field isolates of the pandemic Eurasian lineage. While 100% of identity was predicted at the amino-acid level, only a single synonymous substitution at position 627 appeared, changing the codon GTG for GTA in the African isolates MAL/19 Karonga and Tanzania/Rubwa/2017/1. Interestingly, this single-nucleotide polymorphism (SNP) might be used as a marker for future molecular epidemiology analyses to differentiate between viruses associated with the Eurasia lineage circulating in Africa and other parts of the world.

The EP296R gene encodes a DNA repair apurinic/apyrimidinic endonuclease of 296 amino acids in length [12]. The comparison among representative isolates indicated that 261 out of 296 sites appeared totally conserved. A total of 35 residues accounted for the variability of the EP296R protein (Figure 1A). Critical residues have been identified among positions 45, 81, 271, and 285 (AP site pocket), 48, 49, 50, 51, 52, 81, 82, 84, and 87 (DNA interaction), and 78, 115,142, 179, 182, 218, 231, 233, and 271 (metal-binding active site). Furthermore, on the basis of differences between isolates and supported by phylogenetic analysis, we were able to group representative isolates in three different clusters (Figure 1B). In this context, the Georgia 2007/1 isolate, representative of the pandemic Eurasian lineage appeared grouped with multiple isolates from groups I and XX, since the amino-acid identity within this group was about 98.87%. Conversely, an amino-acid identity around 95.36% and 94.45% was predicted between group I and groups II and III respectively, reflecting the differences between Georgia 2007/1 isolate and isolates form these groups. An additional finding was the identification of residues 125 and 261, in which substitutions V–M and I–V appeared as a putative marker of isolates from the Eurasian lineage (Figure 1A). As described above, these residues might support future epidemiological analysis to differentiate between isolates from the Eurasian isolate and isolates from other genetic sources.

**Figure 2:**
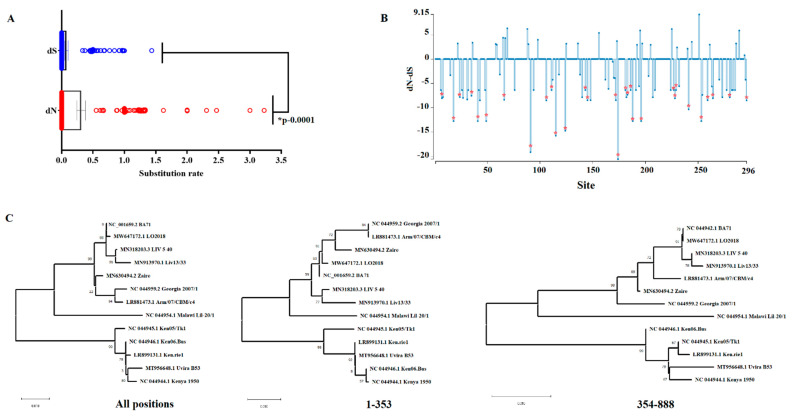
Evolutionary signatures of EP296R gene of ASFV. (**A**) Comparison between synonymous (dS) and nonsynonymous (dN) substitution rates during the evolution of EP296R gene. Significant differences between means at dS and dN were determined by the unpaired *t*-test. Analysis was conducted on the software Jalview version 2.11.1.7. (**B**) Graphic representation of the ratio dN-dS at specific codon sites in the EP296R gene of ASFV. Red asterisks represent codon sites evolving under purifying selection. Analysis was conducted using the evolutionary algorithms FEL considering a cutoff value of *p* = 0.1. (**C**) Phylogenetic analyses using different segments (all sites, 1–353 and 354–888) of the EP296R gene of ASFV were conducted by the neighbor-joining method and the *p*-distance model to show the topology incongruency produced by the recombinant breakpoint predicted by GARD at nucleotide 353. Numbers above internal branches represent bootstrap values (1000 repetitions). Phylogenetic analysis was conducted using the software MEGA 10.2.5.

Lastly, to get a better understanding about the evolutionary signatures of EP296R, we conducted a systematic evolutionary analysis as previously described [19]. First, using the single-likelihood ancestor counting algorithm (SLAC) [20], we obtained the synonymous (dS) and nonsynonymous (dN) substitution rates at different codon sites in the alignment. Overall, our results showed that during the evolution of EP296R gene, synonymous mutations (0.311) were significantly (*p* < 0.05) fixed four times faster than nonsynonymous ones (0.077) (Figure 2A). This fact was consistent with the overall dN/dS rate ratio = 0.233, indicating that purifying selection is the main evolutionary force driving the evolution of EP296R gene. The latter was supported by the results obtained by the algorithm fixed effects likelihood (FEL) [20], where 33 codon sites were found under negative selection (codons 7, 18, 24, 27, 31, 35, 41, 49, 66, 91, 106, 111, 115, 124, 143, 145, 148, 172, 174, 181, 183, 186, 188, 196, 227, 228, 229, 241, 253, 260, 264, 280, and 296) (Figure 2B). Although just sites 49 and 115 were associated with previously described critical sites at DNA interaction and metal-binding active sites of this protein, the remainder of the sites identified by FEL may represent potential functional sites preserved during the evolution of this protein. The identification of these sites may represent a framework for future studies on this protein.

No evidence of positive selection was predicted by FEL or the mixed-effects model of evolution (MEME) algorithm [21]. Multiple nonsynonymous mutations impacting the evolution of codon sites at rates of dN-dS >1 (Figure 2B) were classified as neutral by FEL, suggesting that amino0acid changes at these residues do not represent an adaptative advantage for the EP296R protein. Interestingly, evidence of recombination was found by the genetic algorithm for recombination detection (GARD) [22]. A single breakpoint was identified by GARD at nucleotide 353 with a high average model support of 0.9853. This inference was supported by an improvement in the score c-AIC = 4075.49 of the models supporting the existence of a break point at position 353, when compared with the score AIC-c = 4103.30 of the model assuming no recombination. These results were consistent with the phylogenetic incongruency produced when compared to tree topologies produced by segments of nucleotides 1–353 vs. 354–888 (Figure 2C). Additionally, the incongruency between topologies was consistent with a correlation of *R^2^* = 0.8759 between both topologies, suggesting that recombination plays a role in promoting the genetic diversity of EP296R gene of ASFV.

### 3.2. Detection of EP296R Transcription

**Figure 3:**
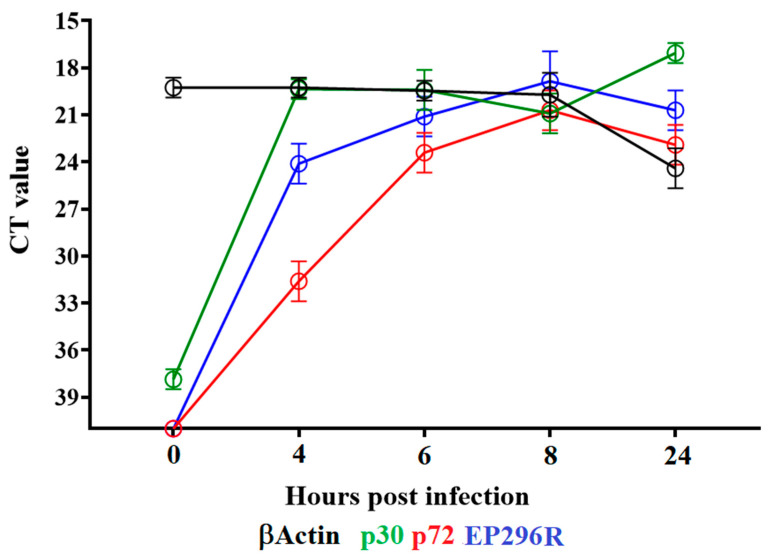
Expression profile of EP296R gene of ASFV during in vitro infection of porcine macrophages. Reverse transcription followed by qPCR was used to evaluate the expression profile of the EP296R gene during in vitro infection at different time points, up to 24 h. As a reference for this analysis, we used qPCRs to specifically detect the expression of genes encoding ASFV proteins p30 (early expression) and p72 (late expression). Additionally, the β-actin gene was used as a control to evaluate the quality and levels of RNA during the infection at different timepoints.

To determine when the EP296R gene is transcribed during the replication cycle, a time-course experiment was performed to analyze the kinetics of RNA transcription in primary swine macrophages infected with ASFV strain Georgia. Swine macrophage cultures were infected at an MOI = 1 with ASFV-G, and cell lysate samples were taken at 4, 6, 8 and 24 hpi. The presence of EP296R RNA was detected by two-step RT-PCR, as described in Section 2. Transcription of EP296R was clearly detected at 4 hpi and remained stable until 24 hpi (Figure 3). The patterns of expression of the well-characterized ASFV early protein p30 (CP204L) and the late protein p72 (B646L) were previously described and are used here as a reference for early and late transcription profiles, respectively. The expression of EP296R followed the pattern of the CP204L gene, suggesting that it is transcribed as an early protein and continues to be transcribed during the virus replication cycle.

### 3.3. Development of the ASFV-G-ΔEP296R Deletion Mutant

**Figure 4:**
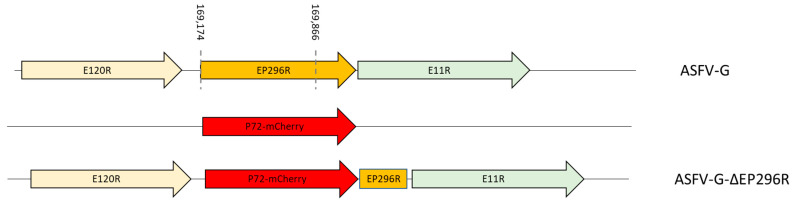
Development of ASFV-G-∆EP296R. The recombinant vector composed by the p72 promoter and a mCherry cassette and the gene positions are indicated. The homologous arms were designed to have flanking ends to both sides of the deletion/insertion cassette. The nucleotide positions of the deleted area are shown by the dashed lines. The resulting ASFV-G-∆EP296R virus is shown on the bottom.

The high level of nucleotide and amino-acid conservation of the EP296R gene among different ASFV isolates and along with its predicted endonuclease function would indicate that EP296R should play an important role in the process of virus replication. To assess the role of the EP296R gene in virus replication both in vitro, in primary swine macrophages cultures, and in vivo, in experimental infected pigs, a recombinant virus harboring a deletion of the EP296R gene was developed (ASFV-G-∆EP296R) using the highly virulent ASFV Georgia 2007 isolate (ASFV-G) as the parental virus. The EP296R gene was replaced with the p72mCherry∆EP296R cassette by homologous recombination with the genome of the ASFV-G. The genome of ASFV-G-∆EP296R presents a deletion of 693 bp (comprised between nucleotide positions 169,174 and 169,866) and substitutes the EP296R gene with a 1226 bp cassette containing the p72mCherry construct (Figure 4). The deletion was designed to leave intact the promoter for neighboring gene E111R, as a full deletion of the EP296R gene would also delete completely the promoter sequence for the E111R gene likely effecting the expression of E111R. Therefore, the 198 nucleotides at the carboxyl end of the EP296R gene remained in the genome of ASFV-G-∆EP296R. Thus, the two functional enzymatic motifs of the EP296R protein (residues 46–58 and 144–149) [12] are absent in the modified version of the ASFV-G-∆EP296R genome. In addition, the remaining carboxyl portion of EP296R gene has no promoter; therefore, it is unlikely to be expressed (Figure 4). The recombinant ASFV-G-∆EP296R was purified after successive limiting dilution steps using primary swine macrophage cell cultures.

The accuracy of the genomic modifications involved in the development of the ASFV-G-∆EP296R genome was evaluated by obtaining its full genome sequence by NGS using an Illumina NextSeq^®^ 500 (San Diego, CA, USA). A total of 816,988 reads were obtained that aligned to the ASFV-G genome. Results demonstrated a 146-nucleotide deletion along with the insertion of 1226 nucleotides, which corresponds to the inclusion of the p72-mCherry cassette. No undesirable significant genomic changes were identified in the ASFV-G-∆EP296R genome. In addition, NGS data showed the absence of parental ASFV-G genome as a potential contaminant in the ASFV-G-∆EP296R stock.

### 3.4. Replication of ASFV-G-∆EP296R in Primary Swine Macrophages

**Figure 5:**
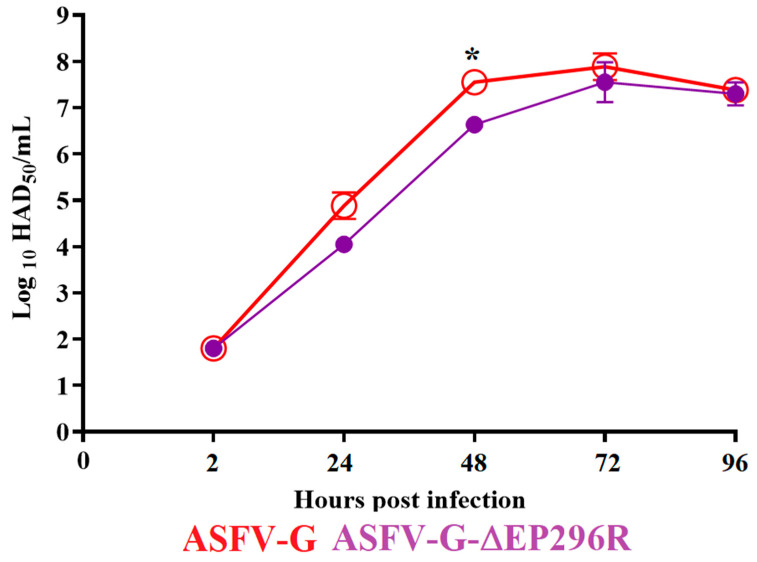
In vitro growth kinetics in primary swine macrophage cell cultures for ASFV-G-∆EP296R and parental ASFV-G (MOI = 0.01). Samples were taken from three independent experiments, at the indicated timepoints, and titrated in swine macrophages. Data represent the means and standard deviations of three replicate wells. Sensitivity using this methodology for detecting virus was ≥log_10_ 1.8 HAD_50_/_mL_. Unpaired *t*-test with Welch correction using the two-stage step-up (Benjamini, Krieger, and Yekutieli) method was conducted to assess statistical differences in viral yields between ASFV-G and ASFV-G-∆EP296R at different hours post infection. Significant differences between viruses were found at 48 h (*) post infection. The significance of this discovery was evaluated using the false discovery rate method (FDR), with *p*-values <0.05 considered significant.

To understand the consequences of the deletion of the EP296R gene during the process of ASFV-G replication, the kinetics of replication of ASFV-G-∆EP296R in primary swine macrophages was assessed using a multistep growth curve. Swine macrophage cultures were infected with an MOI of 0.01 with either ASFV-G-∆EP296R or ASFV-G, and virus yields were assessed at 2, 24, 48, 72, and 96 h post infection (pi). Results demonstrated that ASFV-G-∆EP296R displayed growth kinetics almost indistinguishable from that of the parental ASFV-G (Figure 5). Only small statistical differences were found in virus titers at 48 h pi, whereby the ASFV-G-∆EP296R titer was slightly lower than that of the ASFV-G parental virus. Therefore, deletion of EP296R gene does not affect the replicative ability of the ASFV in swine macrophages.

### 3.5. Assessment of ASFV-G-∆EP296R Virulence in Swine

**Figure 6:**
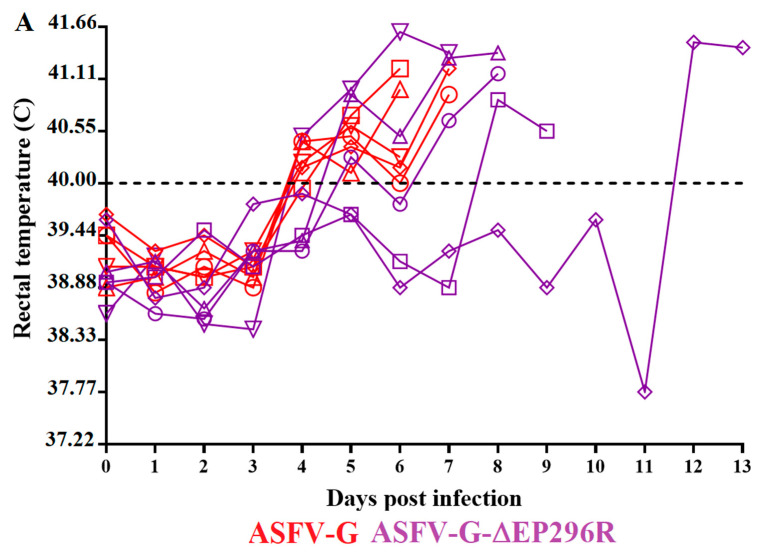
Evolution of body temperature in animals (five animals/group) IM infected with 10^2^ HAD_50_ of either ASFV-G-∆EP296R or parental ASFV-G. No significant differences in the evolution of body temperature were found between groups when analyzed by the unpaired *t*-test using the two-stage step-up (Benjamin, Krieger, and Yekutieli) method.

**Figure 7:**
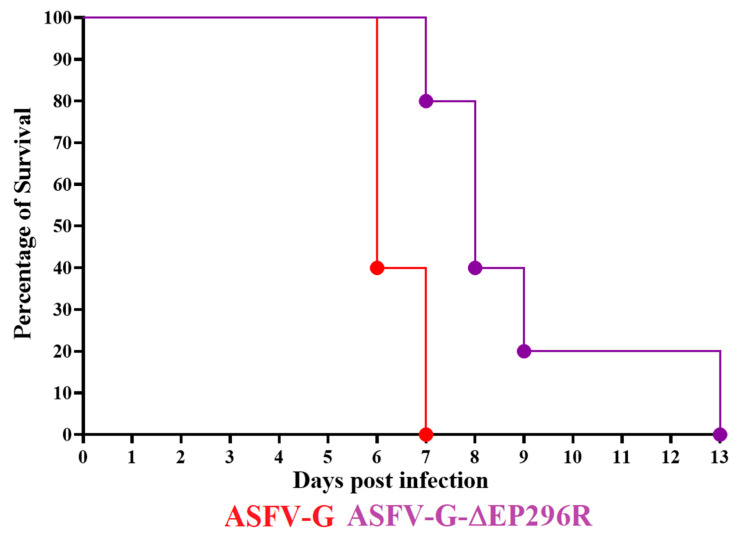
Evolution of mortality in animals (five animals/group) IM infected with 10^2^ HAD_50_ of either ASFV-G-∆EP296R or parental ASFV-G. Significant statistical differences were predicted between the two groups of pigs when evaluated by both the log-rank (Mantel–Cox) test (*p*-value = 0.008) and the Gehan–Breslow–Wilcoxon test (*p*-value = 0.009).

Once tests established that ASFV-G-∆EP296R replicated in swine macrophages cultures as efficiently as the parental virus ASFV-G, it was important to evaluate the effect of deleting the EP296R gene from the genome of ASFV-G in virulence in swine. An experiment involving two groups of 80–90 lb. domestic pigs (five per group) was performed, which evaluated the evolution of disease following experimental infection with either ASFV-G-∆EP296R or ASFV-G. Animals were inoculated IM with 10^2^ HAD_50_ of either virus. The clinical evolution of that group was monitored for 28 days. All animals inoculated with virulent ASFV-G showed a disease onset with an increase in body temperature (over 40 °C) by day 4 pi, followed by the development of a full clinical disease (depression, anorexia, staggering gait, diarrhea, and purple skin discoloration) with all animals being euthanized by day 6–7 pi ( Figure 6 and Figure 7).

On the other hand, some of the animals inoculated with ASFV-G-ΔEP296R showed a lethal, but protracted, clinical form of the disease. Three of the animals started showing an increase in body temperature by day 4–5 pi, followed by a full presentation of clinical disease and euthanasia on day 7 or 8 pi. The other two animals remained clinically normal until days 8 and 12 when they presented a sudden increase in body temperature and a quick evolution of the disease, being euthanized on days 9 and 13 pi, respectively. Significant differences were detected in the patterns of survival curves and temperatures between the two groups of animals ( Figure 6 and Figure 7). Nevertheless, apart from the delay in the outcome, the evolution of the clinical signs of the disease, and the time of euthanasia in animals inoculated with ASFV-G-ΔEP296R, the deletion of EP296R gene did not radically influence the virulence of ASFV-G in domestic pigs.

**Figure 8:**
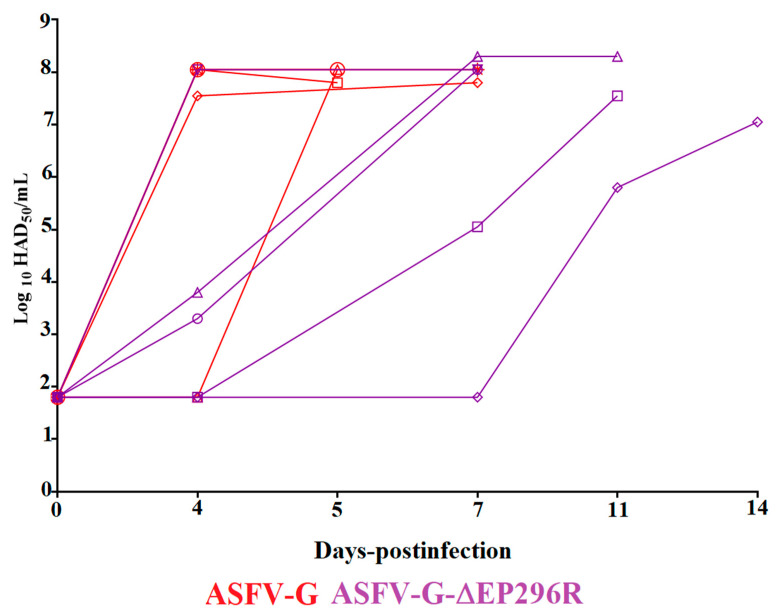
Viremia titers detected in pigs IM inoculated with 10^2^ HAD_50_ of either ASFV-G-∆EP296R, or ASFV-G. Each curve represents individual animal titers. Sensitivity of virus detection: >log_10_ 1.8 TCID_50_/_mL_. Significant differences in viremia titers between the two groups were detected at day 4 post infection, calculated using the unpaired *t*-test considering the two-stage step-up (Benjamini, Krieger, and Yekutieli) method. The significance was evaluated using the false discovery rate method (FDR), with *p*-values <0.05 considered significant. All calculations were conducted using the software GraphPad Prism version 9.2.0.

The kinetics of replication of ASFV-G-∆EP296R in the inoculated animals was assessed by evaluating viremia titers throughout the experimental period and comparing them with that of animals inoculated with ASFV-G (Figure 8). Viremias in animals experimentally inoculated with the virulent parental ASFV-G had high titers (ranging from 10^7.5^ to 10^8^ HAD_50_/_mL_) at day 4 pi in all animals but one, remaining high until the day all four animals were euthanized. The fifth animal presented a sudden increase in virus titers (10^8^ HAD_50_/_mL_) by the day 5 pi, when it was euthanized. Viremias in animals inoculated with ASFV-G-∆EP296R presented similar heterogenous patterns following the evolution of the clinical sign of the disease. One of the animals presented a high viremia titer (10^8^ HAD_50_/_mL_) by day 4 pi, which remained high until the animal was euthanized on day 7 pi. Two other animals showed intermediate viremia titers (10^3.2^–10^3.8^ HAD_50_/_mL_) on day 4 pi, strongly increasing (10^7.5^–10^8^ HAD_50_/_mL_) by day 8 pi, when they were euthanized. Viremia in the fourth animal remained undetectable until day 7 pi when titer increased to 10^4.8^ HAD_50_/_mL_ and further increased to 10^7.5^ HAD_50_/_mL_ at the time of euthanasia, by day 9 pi. The fifth animal also had undetectable viremias until day 9 pi (presenting titers of 10^5.5^ HAD_50_/_mL_) with a further increase to viremia values of 10^6.8^ HAD_50_/_mL_ by the day of euthanasia (day 13 pi). Therefore, viremia titers reproduced the differences in the kinetics of the presentation of the clinical signs of disease in animals inoculated with ASFV-G-∆EP296R and ASFV-G. As in the case of the presentation of clinical disease, some statistical differences among viremias in ASFV-G-∆EP296R and ASFV-G inoculated animals could be observed when virus titers were compared between animals in the two groups.

## 4. Discussion

The results presented in this report show for the first time that the EP296R gene is a nonessential gene that, when deleted, does not affect virus replication in macrophage cell cultures. This result contradicts previous reported data using the ASFV BA71V strain, where deletion of the EP296R gene did severely affect virus replication in swine macrophages [12]. These differences may be attributed to the way that the EP296R gene was deleted. In our study, we left the promoter to E111R intact; thus, it is possible that the deletion in the previous study caused a decrease in expression of E111R, which was the reason for the difference in phenotypes. Alternatively, these differences could be attributed to the genomic differences in the BA71V strain when it was adapted to Vero cells, causing the loss of 11 genes belonging to the MGF360/505 [14]. It is also possible that further adaptation to Vero cells occurred during the process of purification. In our case, ASFV-G was a field isolate not suffering any detectable significant genomic alteration. Our deletion of EP296R also left intact the promoter to the neighboring gene E111R, which could also explain the difference in the observed phenotype. Therefore, phenotypic changes in recombinant ASFV-G-ΔE296R must be directly imputable to the EP296R gene deletion. Perhaps, additional modifications in the genomic background of the parental ASFV BA71V may hamper the substitution of the EP296R gene function, as may be the case in parental ASFV-G.

Our results also demonstrated that deletion of the EP296R gene does not affect the process of virus replication during the infection in domestic swine nor the level of virulence of the ASFV-G. Although statistical differences were established in the protracted viremia kinetics in animals infected with ASFV-G-∆EP296R, compared with those in ASFV-G-infected ones, the biological implication of those differences should be relativized since those animals evolved in all cases toward lethal disease presentation. It is important to mention that NGS analysis of the virus obtained from the euthanized animals showed the exclusive presence of ASFV-G-ΔEP296R, eliminating the possibility that the disease of these animals was caused by the hypothetical presence of residual parental ASFV-G in the ASFV-G-ΔEP296R stock.

All genomes of ASFV have over 150 different proteins. Some individual genes have shown that, when deleted, they are fully attenuated in swine, leading to the development of experimental vaccines [3] or a commercial vaccine now approved for use in Vietnam [2,9]. Similar to the deletion of EP296R, other individual genetic deletions seem to have no effect on virus virulence such as deleting X69R [23] or C9629 [24]. However, understanding what genes can be deleted from ASFV-G is of great importance, as combinations of gene deletions have led to a decrease in virus virulence. For example, the deletion of UK in combination with either 9GL [10] or CD2 [25] resulted in complete attenuation in swine. Individual deletion of UK and CD2 as the deletion of EP296R produced a slight reduction in virus virulence. However, the prediction of attenuation from multiple deletions has not been successful; for example, the deletion of CD2 and EP153R to the experimental vaccine backbone containing a deletion in 9GL [26] abrogated the effectiveness of the experimental vaccine, adding to the complexity of genetic deletions in ASFV. Deletions in the MGF genes have also shown that individual family members can be deleted without effecting the pathogenesis of ASFV [27,28,29] or cause partial attenuation [30] and larger deletions have allowed for further attenuation [6,31] or for the loss of the ability for ASFV to replicate in swine [18], in some cases allowing for growth in continuous cell cultures [32].

Next-generation live-attenuated vaccines will also likely incorporate additional deletions for use as a serological target to differentiate between a previously vaccinated or infected animal; however, deletion of a serological marker has so far reduced vaccine efficacy [33]. It is important to understand which viral genes can be deleted from the ASFV-G, as some genes have been identified as being essential, e.g., EP152R [34], whereas other genes, e.g., KP177R, could be deleted from the ASFV-G isolate [35] but not from other isolates [36]. The importance for identifying genetic deletions in current circulating strains of ASFV is critical information that is necessary for next-generation rationally designed vaccine platforms or for determining the minimal genome for ASFV replication.

## Data Availability

Not applicable.
